# Identification of neoplasm-specific signatures of miRNA interactions by employing a systems biology approach

**DOI:** 10.7717/peerj.14149

**Published:** 2022-10-03

**Authors:** Reza Arshinchi Bonab, Seyedehsadaf Asfa, Panagiota Kontou, Gökhan Karakülah, Athanasia Pavlopoulou

**Affiliations:** 1 Izmir International Biomedicine and Genome Institute, Dokuz Eylül University, Izmir, Turkey; 2 Izmir Biomedicine and Genome Center, Izmir, Turkey; 3 Department of Mathematics, University of Thessaly, Lamia, Greece

**Keywords:** miRNA interactions, Neoplasms, Bioinformatics, Network analysis, Mutual exclusivity, Modules, Diagnosis

## Abstract

MicroRNAs represent major regulatory components of the disease epigenome and they constitute powerful biomarkers for the accurate diagnosis and prognosis of various diseases, including cancers. The advent of high-throughput technologies facilitated the generation of a vast amount of miRNA-cancer association data. Computational approaches have been utilized widely to effectively analyze and interpret these data towards the identification of miRNA signatures for diverse types of cancers. Herein, a novel computational workflow was applied to discover core sets of miRNA interactions for the major groups of neoplastic diseases by employing network-based methods. To this end, miRNA-cancer association data from four comprehensive publicly available resources were utilized for constructing miRNA-centered networks for each major group of neoplasms. The corresponding miRNA-miRNA interactions were inferred based on shared functionally related target genes. The topological attributes of the generated networks were investigated in order to detect clusters of highly interconnected miRNAs that form core modules in each network. Those modules that exhibited the highest degree of mutual exclusivity were selected from each graph. In this way, neoplasm-specific miRNA modules were identified that could represent potential signatures for the corresponding diseases.

## Introduction

MicroRNAs (miRNAs) are endogenous, small (∼22 nucleotides in length), non-protein-coding RNAs that play a critical role in regulating the expression of target mRNAs at the post-transcriptional level by complementary base-pairing of the so-called miRNA “seed” region with the 3′ UTR region of the target (Bartel, 2004; Kehl et al., 2017). In this way, miRNAs reduce the stability of the target mRNA and/or inhibit its translation, thereby downregulating the expression of the corresponding gene (Ambros, 2004).

A single miRNA can potentially regulate the expression of multiple genes, and conversely, a gene can be targeted by numerous miRNAs. Because of this property, miRNAs have been reported to be dysregulated in diverse human diseases (Ardekani & Naeini, 2010; Chung et al., 2021; Climent et al., 2020; Juzwik et al., 2019; Kweon et al., 2020; Lam et al., 2018; Ojha et al., 2019; Sell et al., 2020; Tufekci et al., 2014; Zhao et al., 2016). Due to their prominent role in diseases, miRNAs are regarded as powerful diagnostic and prognostic biomarkers (Condrat et al., 2020; Gandellini, Giovannetti & Nicassio, 2015). As such, miRNA signatures would enable the timely, accurate, non-invasive diagnosis of diverse diseases, including cancers (Song et al., 2017; Zabolotneva et al., 2012).

Several miRNAs may function either as oncogenes (“oncomiRs”) or tumor suppressors (“suppressomiRs”), over- and under-expressed, respectively, in multiple cancers (Zhang et al., 2007). For example, the known oncomiR miR-21 is one of the most frequently upregulated miRNAs in solid tumours (Bautista-Sanchez et al., 2020; Jenike & Halushka, 2021; Wu, Li & Li, 2015), and also members of the miR-34 family are considered as potent tumor suppressive miRNAs (Kalfert et al., 2020; Zhang, Liao & Tang, 2019); MRX34, a liposomal miR-34a mimic has been evaluated in patients with advanced solid tumors (Hong et al., 2020). In addition, miR-143, miR-221/222, and miR-452 are considered potent biomarkers for tumor stratification and diagnosis (Puerta-Gil et al., 2012). Moreover, miRNAs have prognostic potential, as they can predict cancer patient survival (Zhou et al., 2015).

Bioinformatics approaches have been widely used to identify and predict miRNA diagnostic signatures for diverse types/subtypes of cancers (Nunez Lopez et al., 2018; Rehman et al., 2019; Shams et al., 2020; Yang, Zhang & Yang, 2019). However, despite the wealth of studies on cancer-associated miRNAs and the identification of miRNAs that could serve as potential diagnostic biomarkers, these studies have largely focused on certain types/subtypes of cancers. Of note, these miRNA signatures ignore mutual exclusivity, *i.e.,* miRNAs used to detect a particular type/subtype of cancer and not another. To the best of the authors’ knowledge, identification of neoplasm-specific miRNAs that are detected exclusively in a particular type/subtype of cancer is lacking. Therefore, there is an emerging need for identifying core miRNA signatures for characterizing diverse cancers. The findings of this study could be considered towards reducing the cases of spurious cancer diagnosis and improving neoplasm-specific detection by the application of panels of miRNAs exclusive and unique to the major types of cancers.

In the present study, we have employed an integrated bioinformatics methodology for the identification of core sets of miRNAs specific to a particular neoplastic disease, by exploiting relevant data from publicly available resources. Only highly connected miRNA modules displaying significant mutual exclusivity were selected for each group of neoplasms. The component miRNAs of those modules could potentially constitute biomarkers for distinguishing different types of cancers, which could complement and update the existing ones

## Materials & Methods

**Figure 1:**
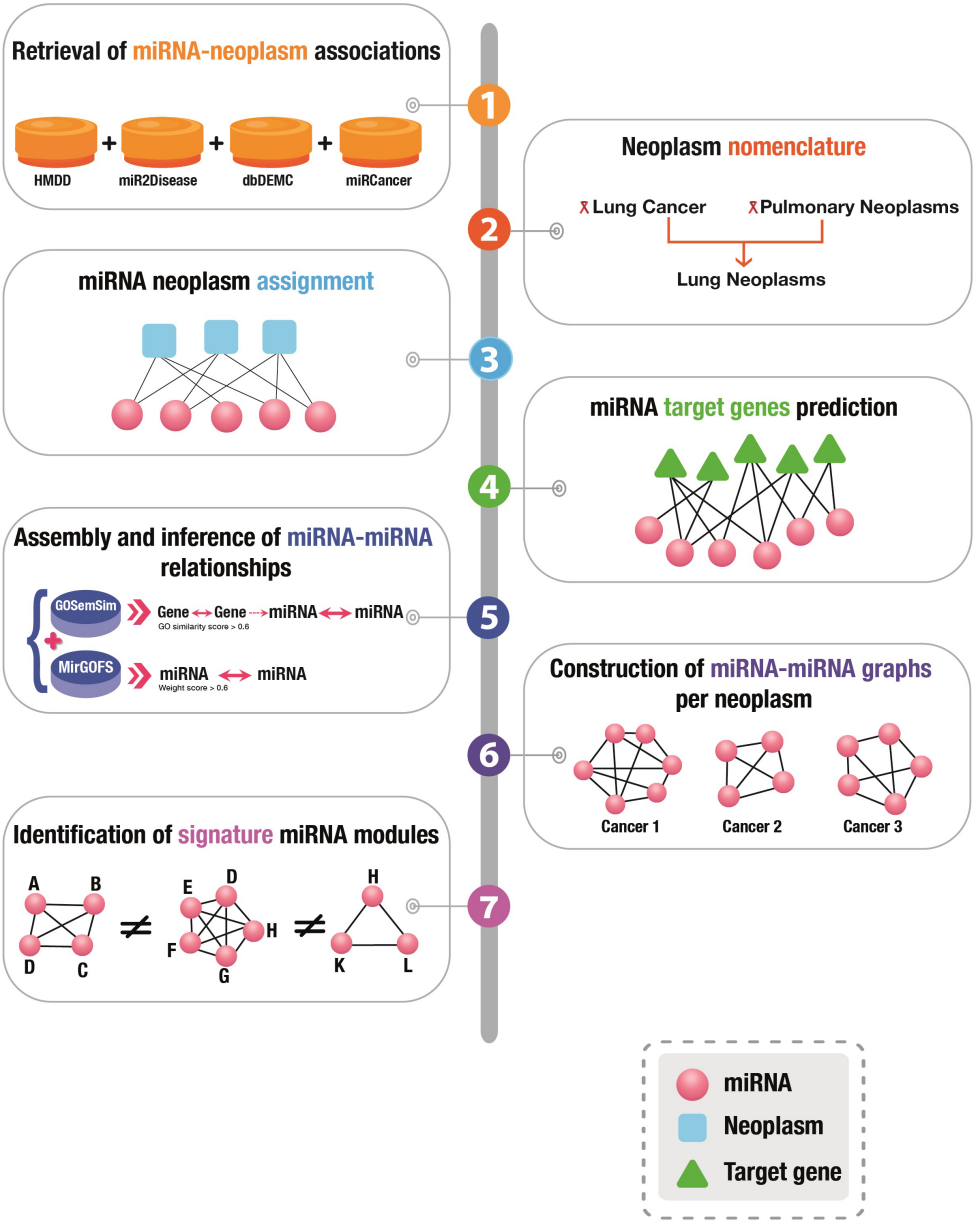
Graphical illustration of the workflow of this study.

A graphical presentation of the overall workflow of this study is depicted in Fig. 1.

All analyses were performed in the R statistical computing environment v.4.2.0 (https://www.r-project.org, accessed on 10 January 2022), unless otherwise stated.

### Acquisition of miRNA-neoplasm associations

The miRNA and neoplasm associations were retrieved from four comprehensive publicly available databases: (a) miR2Disease (Jiang et al., 2009), a manually curated repository of miRNAs deregulated in diverse human diseases, (b) miRCancer (Xie et al., 2013) contains miRNA–cancer association data extracted through literature mining, (c) dbDEMC (Yang et al., 2017), a database of differentially regulated miRNAs in cancers, and (d) HMDD v3.2 (Huang et al., 2019b), a repository for experimentally verified human miRNA-disease relationships. In-house R scripts were used to extract the miRNA-cancer pairwise associations from those databases; duplicate entries were removed.

### Nomenclature

In order to maintain a consistent nomenclature for the neoplasm names from the four databases, the disease terms were annotated according to the International Classification of Diseases and Related Health Problems (ICD), revision 11 (ICD-11) (Pickett & Anderson, 2018). ICD is maintained by the World Health Organization (WHO) and represents the global, comprehensive reference for the collection, processing and hierarchical classification of diseases, which can be used accurately and effectively to investigate the relationships among different diseases. NCBI’s Medical Subject Headings (MeSH) (Lipscomb, 2000), a comprehensive, hierarchically-organized vocabulary for subject indexing, cataloguing and searching biomedical and life sciences-related information, was used for assigning the neoplastic diseases to broader groups.

Official gene symbols were assigned to gene names according to the HUGO Gene Nomenclature Committee (HGNC) (Tweedie et al., 2021); the official miRNA names in miRBase (Kozomara, Birgaoanu & Griffiths-Jones, 2019) were used in this study.

### Prediction of miRNA target genes

The corresponding gene targets of the miRNAs under investigation were identified. To this end, both the experimentally supported and predicted mRNA targets of the neoplasm-associated miRNAs were retrieved with the usage of the following state-of-the-art software tools: (i) microT_CDS has implemented the target prediction algorithm DIANA-microT-CDS (Paraskevopoulou et al., 2013); (ii) TargetScan searches for the presence of conserved sites that match the seed region of each miRNA (Agarwal et al., 2015); (iii) miRDB predicts functional miRNA gene targets by machine learning modeling of target gene expression data (Chen & Wang, 2020; Liu & Wang, 2019); (iv) PicTar relies on an algorithm for the identification of common microRNA targets (Krek et al., 2005); (v) miRanda, which is available online as part of the miRanda-mirSVR tool, depends on evolutionarily conserved miRNA-targets (Enright et al., 2003). To enhance the robustness of the prediction accuracy, only those target genes predicted by more than three tools were included in the study.

### Integration of miRNA-miRNA interactions

The miRNA-miRNA pairwise functional relationships were assembled and inferred from two diverse sources, subsequently followed by merging and removal of duplicate miRNA-miRNA interactions.

GOSemSim (Yu et al., 2010), utilizes information-theoretic measurement to find similarities between genes based on Gene Ontology (GO) (Ashburner et al., 2000) terms. Thus, GOSemSim assigns a function to genes according to shared GO annotations. A miRNA-miRNA matrix was created based on the degree of functional similarity of their target genes; the higher the degree of gene relatedness, the higher the association score of the respective miRNAs.

In addition, miRNA-miRNA pairwise relationships were obtained from MIRGOFs (Yang et al., 2018), which measures functional similarities for miRNAs based on the GO annotation of their corresponding target genes by adopting two novel features: (i) it utilizes a new GO semantic similarity metric which considers both common ancestors and descendants of GO terms; (ii) it computes similarity between GO sets in an asymmetric manner, and weights each GO term by its statistical significance.

Only those miRNA pairwise interactions, from GoSemSim and MIRGOFs, with weight score ≥ 0.6 (where “1” is the highest) were selected to ensure robustness.

### Construction of miRNA-neoplasm networks

Firstly, miRNA-neoplasm networks were constructed for each group of neoplasms, where the links were assigned two weights, one from MIRGOFs (Yang et al., 2018) and the second one from the calculated miRNA matrix based on GOSemSim.

The topological features of the generated networks were investigated through Cytoscape (v.3.9.1) (Shannon et al., 2003), a software platform for network manipulation and visualization. Arena3Dweb, an interactive, web-based tool for the visualization of multilayer networks in tree-dimensional space was also used for network depiction (Karatzas et al., 2021).

### Identification of mutually exclusive cancer-relevant miRNA modules

To achieve the maximum possible mutual exclusivity, first, unique miRNAs were detected in each neoplasm. Second, the *igraph* package in R was used to calculate the node degree distribution (Barabasi, Gulbahce & Loscalzo, 2011) and clustering coefficient (*i.e.,* an estimate of the tendency of the nodes in a graph to cluster together) (Watts & Strogatz, 1998) in each miRNA-neoplasm network (a total of thirty graphs). Then, the degree distribution was applied and a simultaneous comparison was performed between the 30 networks in order to find modules of miRNAs that are included in one neoplasm and not in the others. An in-house R script was used in order to handle the complexity of the datasets. Every dataset was included in a *hash* and the mutually exclusive modules were detected.

## Results

Functionally linked diseases tend to share common epigenetic mechanisms to ensure the coordinated regulation of their causative genes (Zinani, Keseroglu & Ozbudak, 2022). MiRNAs cooperate towards gene co-targeting and co-regulation in various neoplastic diseases, and hence, tend to form densely connected communities in the disease regulome (Bryan et al., 2014). Pivoted on this premise, the aim of this study was to explore the miRNA-miRNA interaction networks for diverse groups of neoplastic diseases, based on the functional relations of their corresponding target genes, towards the discovery of core sets of miRNAs per cancer that could represent potential signatures for each cancer type. To this end, this work sought to identify tightly interconnected modules per neoplasm with the highest possible degree of mutual exclusivity based on network topological properties.

### Neoplasm classification

All neoplasms were classified into thirty major groups using first the ICD-11 standard hierarchy for medical terms and then the MeSH vocabulary for assigning those terms to broader classes (Table 1). For example, the ICD terms ‘adenocarcinoma of lung’, ‘follicular carcinoma of thyroid gland’, and ‘adenoid cystic carcinoma’ were classified under the broader MeSH term ‘adenocarcinoma’. The ambiguous terms were removed from the subsequent stages of the analysis.

### Construction of miRNA-miRNA graphs per neoplasm

The miRNA-neoplasm bipartite graphs were constructed by relying entirely on experimentally known miRNA-cancer associations assembled and integrated from four diverse resources.

The miRNA-miRNA interactions in the corresponding thirty monopartite networks were inferred from two different sources, directly and indirectly. In the former case, the pairwise miRNA associations were obtained from miRGOFS which includes miRNA-miRNA similarities computed with respect to the functional relationships of their target genes according to shared GO terms. In the latter case, miRNA targets were first detected through the consensus output of more than three miRNA-gene interactions databases, which include both experimentally supported and predicted entries. Then, GOSemSim was deployed, where the similarities for each pair of miRNAs were computed based on the functional annotation of their target genes according to GO; the more semantically related two GO terms, the higher the degree of association of the corresponding genes. The miRNA-miRNA pairs from both sources were combined and the duplicates were removed; a total of 2604 unique miRNAs were detected across the thirty neoplasms (Table S1).

**Table 1:** Major classes of neoplasms, neoplasm-associated miRNAs and miRNA-miRNA interactions.

Neoplasm	Unique miRNAs	miRNA pairs
Adenocarcinoma	551	51,455
Adrenal Gland Neoplasms	431	22,150
Biliary Tract Neoplasms	1,050	147,665
Brain Neoplasms	1,679	267,717
Breast Neoplasms	1,762	288,200
Colorectal Neoplasms	1,821	304,757
Endocrine Gland Neoplasm	39	241
Esophageal Neoplasms	1,806	301,059
Eye Neoplasms	125	2,885
Head and Neck Neoplasms	1249	155,197
Hematologic Neoplasms	54	492
Intestinal Neoplasms	403	28,209
Kidney Neoplasms	1,252	152,880
Leukemia	286	15,275
Liver Neoplasms	1,463	266,584
Lung Neoplasms	1,736	282,743
Lymphoma	1,330	177,172
Nerve Tissue Neoplasms	211	7,005
Ovarian Neoplasms	1,404	245,655
Pancreatic Neoplasms	1,701	272,487
Pleural Neoplasms	112	2,928
Prostatic Neoplasms	1,110	164,209
Sarcoma	1,378	235,334
Skin Neoplasms	1,197	143,565
Squamous Cell Carcinomas	884	118,173
Stomach Neoplasms	1,660	260,636
Testicular Neoplasms	178	4,690
Thyroid Neoplasms	1,108	126,140
Urinary Bladder Neoplasms	1,528	224,985
Uterine Neoplasms	329	18,475

The miRNAs hsa-miR-101-3p, hsa-miR-145-5p, hsa-miR-223-3p were found in all 30 types of cancers (Table S1). Furthermore, the well-studied oncomir miR-21, as well as the suppressomir miR-34a, were detected in 29 neoplasms, except testicular cancers. Fifteen unique miRNAs were found in ten neoplasms, namely, brain (hsa-miR-291-5p), colorectal (hsa-miR-103a-1-5p), head and neck (hsa-miR-6836-3p), liver (hsa-miR-199a-1-5p, hsa-miR-199a-2-5p), lung (hsa-miR-886-5p, hsa-miR-886-3p, hsa-mir-320a-3p), ovarian (hsa-miR-526a-5p), skin (hsa-miR-3689c), stomach (hsa-miR-7156-3p), thyroid (hsa-miR-5686, hsa-miR-199a-3p, hsa-miR-199b-3p), urinary bladder (hsa-miR-498-3p).

All thirty networks are very dense with an average estimated clustering coefficient ∼0.89 (where ‘1’ is the highest value). This indicates that the nodes in the given graphs exhibit a high tendency to cluster together. Despite the stringent cutoff values, the number of significant links is quite large, and as such, no visually discernible modules could be detected in any of the networks. Based on topological analyses, all graphs have a random degree distribution, where the node degrees (*i.e.,* the number of connections of a given node to neighboring nodes) follow a Poisson distribution, unlike the biological networks which are scale-free and are dominated by few ‘hubs’ (*i.e.,* highly connected nodes) (Barabasi, 2009; Barabasi, Gulbahce & Loscalzo, 2011; Goh et al., 2007; Kontou et al., 2016). This is probably attributed to the “promiscuous” nature of miRNAs to potentially target numerous genes, resulting in this way to a high number of corresponding miRNA connections.

**Figure 2:**
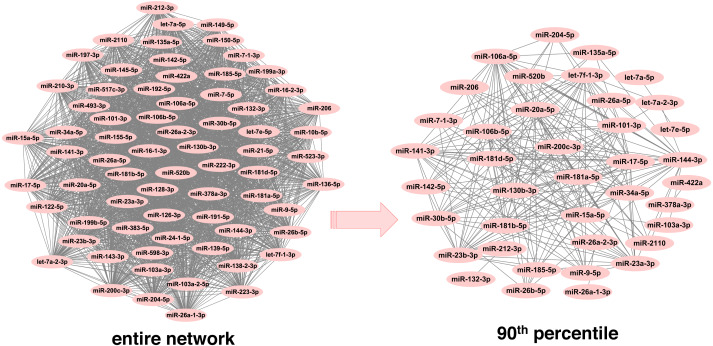
Endocrine gland neoplasm miRNA-miRNA graph. The nodes represent miRNAs and the connecting lines indicate the functional relations between miRNAs. In the original network (left) there are 70 nodes and 2,415 pairs, whereas in the pruned/filtered network (right) there are 39 nodes and 241 links.

In order to infer less dense and more robust networks, only those links with weights above the 90th percentile per network were selected. In this way, the highest scoring miRNA-miRNA interactions were used for network construction and subsequent module detection. After pruning, there were 1,893 unique miRNAs across neoplasms, with the number of miRNAs per neoplasm ranging from 39 (endocrine gland neoplasm) to 1,821 (colorectal neoplasms). Consequently, the number of the corresponding pairwise miRNA-miRNA associations was reduced significantly (Table 1 and Table S2). Noteworthy, this is observed in a more pronounced way in the more populated graphs, since the number of potential miRNA connections increases exponentially with an increase in the number of member miRNAs. As it is shown in Fig. 2, the endocrine gland neoplasm miRNA network becomes less dense with a reduced number of nodes (miRNAs) and connections (miRNA relations) when the upper 10th percentile of miRNA-miRNA links was used for graph construction.

### Detection of neoplasm-specific miRNA modules

The goal of this study was to detect distinct subgraphs within the neoplasm graphs which cannot be contained by another subgraph. These subgraphs, which represent local clusters or modules, are considered semiautonomous and therefore are of high biological importance (Pelaez & Carthew, 2012). The top scoring mutually exclusive modules per neoplastic disease were selected based on the criteria that they exhibited significant mutual exclusivity and their constituent miRNAs were more highly connected to each other than to other miRNAs in the entire graph.

**Figure 3:**
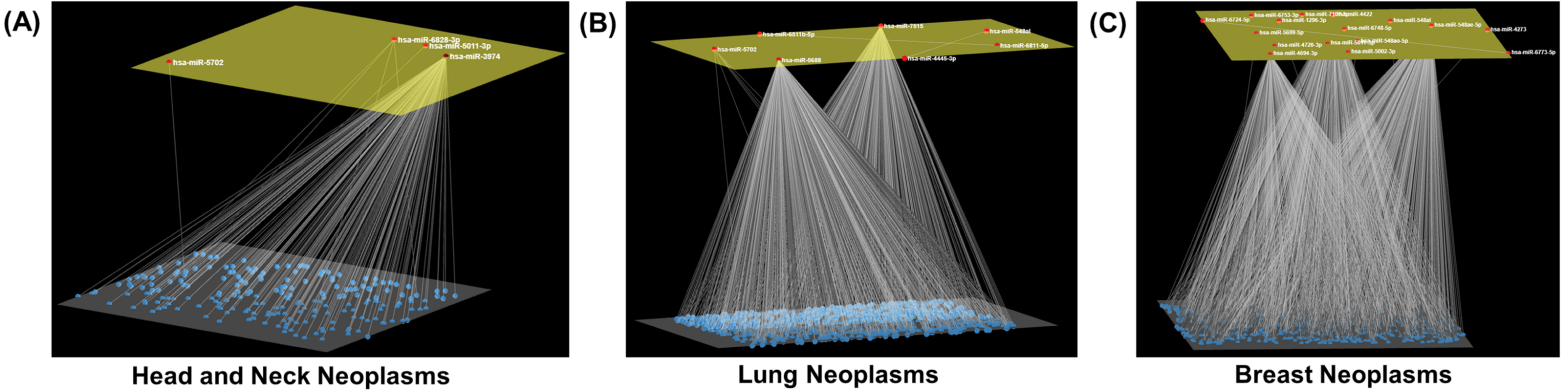
Neoplasm-specific modules. Depiction of modules consisting of a (A) small (head and neck neoplasms), (B) moderate (lung neoplasms), and (C) large number (breast neoplasms) of miRNAs. The module miRNAs are shown in the upper layer, and their associations, both between them and with other miRNAs in the neoplasm network, are displayed in the lower layer. Arena3D was used for visualization.

Modules of miRNA interactions that displayed a statistically significant level of mutual exclusivity were detected in seventeen neoplasms (Table 2). A single miRNA-miRNA pair (*i.e.,* hsa-miR-1296-3p and hsa-miR-6748-5p) was detected in bladder cancer, which was excluded from the analysis. In the other twelve neoplasms under study, no distinguishable modules could be detected. The seventeen modules were comprised of three to twenty-three miRNAs (Table 2). Of note, only a few miRNAs were found in up to five cancer modules (hsa-miR-4273, hsa-miR-4422, hsa-miR-4720-3p and hsa-miR-5002-3p); this is because mutual exclusivity was a prerequisite. Among them were modules consisting of a relatively large number of miRNAs, such as the esophageal (23), colorectal neoplasms (20), and breast neoplasms (17), and also modules with a moderate number of component miRNAs including the liver (8) and lung neoplasms (7). Cliques of only three miRNAs were detected in the sarcoma and squamous cell carcinomas (Fig. 3). In addition, PubMed (https://pubmed.ncbi.nlm.nih.gov/) was searched with each of the neoplasms and the core miRNA components of the corresponding modules (Table 2). Nonetheless, in spite of extensive searches, any reported combinations of any two miRNAs that compose the neoplasm-specific modules could not be detected (Table 2).

**Table 2:** Mutually exclusive miRNA modules per neoplasm.

Neoplasm	miRNA modules
Brain neoplasms	hsa-miR-3689e, hsa-miR-4422, hsa-miR-4432, hsa-miR-4720-3p, hsa-miR-5002-3p, hsa-miR-548ao-5p, hsa-miR-5688, hsa-miR-6511b-5p, hsa-miR-6719-3p, hsa-miR-6753-3p, hsa-miR-6811-5p, hsa-miR-7107-3p, hsa-miR-8062, hsa-miR-8067, hsa-miR-8068
Breast neoplasms	hsa-miR-1296-3p, hsa-miR-4273, hsa-miR-4422, hsa-miR-4445-3p, hsa-miR-4694-3p, hsa-miR-4720-3p, hsa-miR-5002-3p, hsa-miR-5011-3p, hsa-miR-548ae-5p, hsa-miR-548al, hsa-miR-548ao-5p, hsa-miR-5699-5p, hsa-miR-6724-5p, hsa-miR-6748-5p, hsa-miR-6753-3p, hsa-miR-6773-5p, hsa-miR-7107-3p
Colorectal neoplasms	hsa-miR-4273, hsa-miR-4293, hsa-miR-4422, hsa-miR-4432, hsa-miR-4445-3p, hsa-miR-4694-3p, hsa-miR-4720-3p, hsa-miR-5002-3p, hsa-miR-5011-3p, hsa-miR-548aj-5p, hsa-miR-548al, hsa-miR-6511b-5p, hsa-miR-6724-5p, hsa-miR-6753-3p, hsa-miR-6770-5p, hsa-miR-6773-5p, hsa-miR-6811-5p, hsa-miR-7107-3p, hsa-miR-7154-5p, hsa-miR-8068
Esophageal neoplasms	hsa-miR-1296-3p, hsa-miR-3974, hsa-miR-4273, hsa-miR-4445-3p, hsa-miR-4720-3p, hsa-miR-5002-3p, hsa-miR-548aj-5p, hsa-miR-548al, hsa-miR-548ao-5p, hsa-miR-5688, hsa-miR-5699-5p, hsa-miR-6511b-5p, hsa-miR-6719-3p, hsa-miR-6724-5p, hsa-miR-6748-5p, hsa-miR-6770-5p, hsa-miR-6773-5p, hsa-miR-6811-5p, hsa-miR-6828-3p, hsa-miR-7154-5p, hsa-miR-7515, hsa-miR-8062, hsa-miR-8067
Head and neck neoplasms	hsa-miR-3974, hsa-miR-5011-3p, hsa-miR-5702, hsa-miR-6828-3p
Kidney neoplasms	hsa-miR-1296-3p, hsa-miR-3689e, hsa-miR-4422, hsa-miR-5699-5p
Liver neoplasms	hsa-miR-3689e, hsa-miR-6724-5p, hsa-miR-6773-5p, hsa-miR-7154-5p, hsa-miR-7159-5p, hsa-miR-8062, hsa-miR-8067, hsa-miR-8068
Lung neoplasms	hsa-miR-4445-3p, hsa-miR-548al, hsa-miR-5688, hsa-miR-5702, hsa-miR-6511b-5p, hsa-miR-6811-5p, hsa-miR-7515
Ovarian neoplasms	hsa-miR-4273, hsa-miR-526a-5p, hsa-miR-6770-5p, hsa-miR-7156-5p, hsa-miR-7515
Pancreatic neoplasms	hsa-miR-3974, hsa-miR-4273, hsa-miR-4293, hsa-miR-4694-3p, hsa-miR-522-5p, hsa-miR-523-5p, hsa-miR-5702, hsa-miR-6770-5p, hsa-miR-6828-3p, hsa-miR-7156-5p, hsa-miR-7159-5p, hsa-miR-7515
Prostatic neoplasms	hsa-miR-1296-3p, hsa-miR-522-5p, hsa-miR-5699-5p, hsa-miR-6748-5p
Sarcoma	hsa-miR-5688, hsa-miR-7515, hsa-miR-8067
Skin neoplasms	hsa-miR-3689c, hsa-miR-3689e, hsa-miR-522-5p, hsa-miR-548ae-5p, hsa-miR-548ao-5p, hsa-miR-5688, hsa-miR-6753-3p, hsa-miR-6828-3p, hsa-miR-7107-3p, hsa-miR-7154-5p, hsa-miR-8062, hsa-miR-8068
Squamous cell carcinomas	hsa-miR-4293, hsa-miR-4694-3p, hsa-miR-548aj-5p
Stomach neoplasms	hsa-miR-4720-3p, hsa-miR-5002-3p, hsa-miR-5699-5p, hsa-miR-5702, hsa-miR-6719-3p, hsa-miR-6828-3p
Thyroid neoplasms	hsa-miR-4293, hsa-miR-4422, hsa-miR-5011-3p, hsa-miR-6753-3p, hsa-miR-7107-3p
Urinary bladder neoplasms	hsa-miR-6719-3p, hsa-miR-6724-5p, hsa-miR-6773-5p, hsa-miR-8067

## Discussion

MicroRNAs constitute major components of the epigenetic regulome of diseases including cancers. Previous studies have focused on the identification of disease-specific miRNA-interaction signatures. Nalluri and colleagues (2017) introduced miRsig, a network inference method for identifying preserved miRNA-miRNA interactions across different types of cancers using miRNA expression profiles (Nalluri et al., 2017). In another study, cancer-specific miRNAs were detected in miRNA target-deregulated networks built on the differentially expression profiles of miRNAs and mRNAs (Xu et al., 2011). Song et al. (2015) identified co-regulating miRNAs in lung cancer based on shared functionally linked target genes. However, these studies have not taken into consideration the mutual exclusivity relations between the neoplastic diseases under investigation.

In the present study, *a priori* miRNA-cancer relationships were collected from four comprehensive databases which contain different categories of association data, *i.e.,* differential expression profiles, circulating biomarkers, genome-wide association studies (GWAS), knockdown studies, etc. This sort of ‘pluralistic’ data would enable the extraction of the maximum amount of available cancer-associated miRNA information, in order to predict and prioritize pairwise miRNA interactions per neoplasm towards the identification of distinct groups of miRNAs. The neoplasm terms were first homogenized, in order to eliminate any duplicate entries. Then, miRNA connections were inferred by relying on the shared functionality of their target genes. Based on the premise that miRNAs contribute to the post -transcriptional gene regulation in a synergistic manner (Shi et al., 2013), by co-ordinately regulating target genes, we generated miRNA-oriented networks of the top-ranking miRNA pairs, and sought to discover modules of tightly connected miRNAs per neoplasm.

Herein, mutual exclusivity was included as a prerequisite in order to detect sets of densely connected miRNAs that could represent the signature component for a certain type of cancer and not for another. This approach would be particularly useful for the accurate detection of specific types of cancers. Likewise, in a prominent study by Ciriello et al. (2012), mutual exclusivity analysis of diverse genomic alterations was performed in order to identify oncogenic pathway modules.

The environmental context, that is, the connection patterns of the individual miRNAs, is very important, since in different niches (*e.g.*, normal *versus* diseased state) the same miRNA may behave differently, including its differential expression status and connections. Therefore, both the miRNAs *per se* and the connection patterns among the component miRNAs of a module should be taken into consideration when extracting miRNA signatures. This is suggestive of the cooperativity between the respective miRNAs, so as to exert their regulatory effect on their targets.

In this study, miRNA modules were identified in seventeen neoplastic disease groups, composed of three to twenty-three members. The unique miRNA hsa-miR-3689c was found in the skin neoplasms module, and was also shown to be overexpressed in vulvar squamous cell carcinoma (Yang & Wu, 2016). Moreover, another unique miRNA, miR-526a-5p, was included in the module of ovarian neoplasms, and was found to be significantly differentially expressed in ovarian cancer (Penyige et al., 2019) (**Table 2**). Of the signature miRNAs (**Table 2**), hsa-miR-6770-5p, is suggested to have prognostic value in triple-negative breast cancer (TNBC) (Wu, Ding & Lin, 2020), and hsa-miR-1296-3p was found to be downregulated in prostate cancer (Majid et al., 2010). In addition, genetic variation in the target site of miR-4273-5p (Table 2) was associated with colorectal cancer susceptibility (Lee et al., 2016).

Of particular note, members of the let-7 (let-7a, 7b, 7c, 7d, 7e, 7f, 7g, 7i, miR-98, and miR-202) (Ma et al., 2021; Mizuno, Kawada & Sakai, 2018), miR-10 (miR-10a/b, miR-99a/b, miR-100 and miR-125) (Lund, 2010; Tehler, Hoyland-Kroghsbo & Lund, 2011), miR-15 (miR-15a/b, miR-16-1, miR-16-2, miR-195, and miR-497) (Aqeilan, Calin & Croce, 2010; Liu et al., 2019), miR-183 (miR-96, miR-182, and miR-183) (Dambal et al., 2015; Ma et al., 2016; Pavlakis et al., 2017), miR-200 (miR-200a, 200b, 200c, miR-429, and miR-141) (Cavallari et al., 2021; Chen & Zhang, 2017; Huang et al., 2019a), miR-221/222 (Garofalo et al., 2012; Song et al., 2019; Zhang et al., 2018), miR-23 (miR-23a, miR-27a, and miR-24-2) (Chhabra, Dubey & Saini, 2010), miR-29 (miR-29a, 29b, 29c) (Jiang et al., 2014; Kwon et al., 2019; Yan et al., 2015), miR-30 (miR-30a, 30b, 30c, 30d, 30e) (Song et al., 2020), miR-34 (miR-34a, 34b, 34c) (Agostini & Knight, 2014; Hermeking, 2010; Kalfert et al., 2020; Zhang, Liao & Tang, 2019), miR-99 (miR-99a, 99b, and miR-100) (Eniafe & Jiang, 2021) families, miR-17/92 (miR-17, miR-18a, miR-19a, miR-20a, miR-19b-1, and miR-92a-1) cluster or ‘oncomiR-1’ (Concepcion, Bonetti & Ventura, 2012; Mendell, 2008), and miR-21 (Bautista-Sanchez et al., 2020; Jenike & Halushka, 2021; Wu, Li & Li, 2015), which are well-documented to be associated with multiple types/subtypes of cancers, occur with a high frequency across all neoplasms (Table S1 ). Of particular note, none of those miRNAs were detected in any of the neoplasm-specific signature modules in **Table 2**. Due to their omnipresence in the major neoplastic diseases, the above miRNAs cannot represent signature components for specific types of cancers. Moreover, similar miRNA signatures were not detected despite thorough literature searches, further highlighting the importance of the findings of this study, as novel miRNA-miRNA interactions related to groups of neoplastic diseases were discovered.

## Conclusion

Taken together, in the present study, a novel computational pipeline is provided, which could likely update and complement previous studies on cancer-relevant miRNAs. The predicted miRNA-miRNA interactions found in the modules of the neoplastic diseases could pave the way for the rational design of experimental studies directed towards the elucidation of these novel pairings. These miRNA-interaction signatures unique to each type of cancer could be possibly utilized in the clinical setting, along with the biomarkers that are currently being applied, for enhancing diagnostic accuracy in differentiating between cancer types; thereby, a panel of miRNAs could be used for the precise detection of a particular neoplastic disease and not another. The overall methodology proposed in this study could be also extrapolated to other datasets for the diagnosis and prognosis of human diseases/disorders.

##  Supplemental Information

10.7717/peerj.14149/supp-1 Supplemental Information 1 List of miRNAs per neoplasm

## Additional Information and Declarations

### Data Availability

The following information was supplied regarding data availability: The list of miRNAs per neoplasm is available in the Supplementary File. The pairwise miRNA-miRNA associations is available at Mendeley Data: Pavlopoulou, Athanasia (2022), “Table S2”, Mendeley Data, V1, doi: 10.17632/cyc6dfgtzs.1.
